# Chemical Composition and Antioxidant Activity of the Main Fruits Consumed in the Western Coastal Region of Ecuador as a Source of Health-Promoting Compounds

**DOI:** 10.3390/antiox8090387

**Published:** 2019-09-10

**Authors:** Mabel Guevara, Eduardo Tejera, María G. Granda-Albuja, Gabriel Iturralde, Maribel Chisaguano-Tonato, Silvana Granda-Albuja, Tatiana Jaramillo-Vivanco, Francesca Giampieri, Maurizio Battino, José M. Alvarez-Suarez

**Affiliations:** 1 Facultad de Ingeniería y Ciencias Aplicadas, Grupo de Investigación en Biotecnología Aplicada a Biomedicina, Universidad de Las Américas, Av. de los Granados E12-41y Colimes esq., EC170125 Quito, Ecuador; 2 Facultad de Ingeniería y Ciencias Aplicadas, Grupo de Bio-Químioinformática, Universidad de Las Américas, Av. de los Granados E12-41y Colimes esq., EC170125 Quito, Ecuador; 3 Laboratorios de Investigación, Universidad de Las Américas, Av. de los Granados E12-41y Colimes esq., EC170125 Quito, Ecuador; 4 Nutrición, Escuela de Salud Pública, Facultad de Ciencias de la Salud, Universidad San Francisco de Quito, Campus Cumbayá, Diego de Robles s/n, 170901 Quito, Ecuador; 5 Departamento de Biotecnología, Universidad de Las Fuerzas Armadas, Túnel Principal Universidad de las Fuerzas Armadas-ESPE, 171103 Sangolquí, Ecuador; 6 Jardín Botánico de Quito, Quito, Interior Parque Carolina, 170135 Quito, Ecuador; 7 Nutrition and Food Science Group, Department of Analytical and Food Chemistry, CITACA, CACTI, University of Vigo—Vigo Campus, 36310 Vigo, Spain; 8 Department of Clinical and Molecular Sciences, Faculty of Medicine, Polytechnic University of Marche, Via Pietro Ranieri, 60131 Ancona, Italy; 9 International Research Center for Food Nutrition and Safety, School of Food and Biological Engineering, Jiangsu University, Zhenjiang 212013, China

**Keywords:** tropical fruits, nutraceuticals, antioxidant capacity, bioactive compounds, Ecuador

## Abstract

We studied 19 different tropical fruits traditionally consumed in the coastal lowlands of Ecuador to determine their chemical composition and antioxidant activity. Carambola (*Averrhoa carambola* L.) had the highest total phenolic, flavonoid, and total antioxidant capacity values, whereas guava fruits (*Psidium guajava* L.) had the highest vitamin C values. The main organic acids identified were lactic, citric, and acetic acids, and the highest amount of lactic acid was found in soursop fruits (*Annona muricata* L.), whereas Ecuadorian ivory palm (*Phytelephas aequatorialis* Spruce) and guava fruits had the highest acetic acid content. Guava also had the highest citric acid content; the highest concentration of oxalic acid was found in carambola. In terms of sugar content, giant granadilla (*Passiflora quadrangularis* L.) had the highest values of glucose, and red mombin (*Spondias mombin* L.) had the largest values for fructose and guava for sucrose. Chili pepper (*Capsicum chinense* Jacq) proved to be the main source of carotenoids, lutein, and β-carotene, anthocyanins, and vitamin C. The results here increase our knowledge regarding the composition of the main fruits consumed on the west coast of Ecuador to facilitate recommendations as potential sources of health-promoting compounds.

## 1. Introduction

The considerable biodiversity found in the South American biomes, such as in Ecuador’s Andean, coastal, and Amazon forest regions, represents a poorly studied source of new plants for human nutrition. Ecuador is one of the most mega-diverse countries in the world, with a natural wealth that distinguishes it from the rest of the Andean countries. The country boasts a large number of fruit specimens, some native to Ecuadorian biomes, with others introduced to the country. These specimens were domesticated by the indigenous native populations over the years, and now occur naturally, especially along the western coastal region of Ecuador [1]. Many of these products are either not well-known or completely unknown, constituting a large number of underexploited native and exotic products that are of potential interest to the agroindustry and as natural sources of bioactive compounds with potential health benefits. Dietary patterns based on the frequent consumption of fruits and vegetables are closely related to a decrease in non-communicable chronic diseases, such as diabetes, cancer [2,3], and Alzheimer’s [4,5], due to their high contents of phytochemicals, vitamins, minerals, and fiber, and to the interactive synergies between these components. Vitamin C (Vit C), a potent antioxidant that is widely found in fruits, is known to be a powerful antioxidant, with implications in several biological functions, such as collagen biosynthesis, L-carnitine, certain neurotransmitters, as well as in the metabolism of certain proteins, and has been shown to regenerate other antioxidants within the body, including α-tocopherol (vitamin E) [6]. Therefore, regular intake is essential to maintain a healthy body and prevent diseases caused by oxidative stress and aging. 

Based on the need to identify new sources of bioactive compounds, the scientific community is actively searching for new sources of bioactive compounds. The consumption of native or exotic fruits from tropical zones has increased due to the recognition of their nutritional and therapeutic values [7,8,9]. Studies on native products, mainly from South America, have also increased, as well as on the development of new food products based on these products; however, in the case of Ecuador, information about their chemical compositions and nutritional values is limited or often nonexistent.

Ecuador is divided into three continental regions: the coast (lowland regions between the Pacific Ocean and the Andes), the mountains (the Andean region), and the Amazon (east of the Andes mountain range), which are characterized by well-defined climatic conditions, vegetation, and nutritional patterns. The coastal region is divided into four provinces—Esmeraldas, Manabí, Guayas, and El Oro—which are characterized by lush vegetation, which contributes significantly to the fruticulture and horticulture of the country. This region is the main tropical fruits production area: native, exotic, or introduced in the country. The fruits are distributed nationwide and, in many cases, exported. Although some reports exist on the composition [10,11,12] and biological effects [7,13] of some fruits traditionally consumed in Ecuador, these results are limited and do not allow us to identify the potential of these products as natural sources of bioactive compounds. These studies have been limited mainly to the profile of a group of bioactive compounds, such as vitamin C and polyphenols, which, although they provide relevant information about the potential of some of these fruits, are not the only important values, since there are other compounds relevant to health and food quality that have not been studied in detail. With this backdrop, the aim of this study was to determine the principal bioactive compounds and antioxidant capacity of the main tropical fruits consumed in the coastal lowlands of Ecuador to elucidate their potential as possible sources of bioactive compounds with potential health benefits.

## 2. Material and Methods

### 2.1. Fruit Samples

We collected 19 fruits in 2018 during their corresponding crop season. They were purchased from popular markets in the coastal lowlands of Ecuador (provinces of Esmeraldas, Manabí, Guayas, and El Oro) (Table 1). Samples without blemishes or damage were selected based on a similar degree of ripeness on two different occasions. Three batches of samples (1 kg) were randomly collected on each occasion in each popular market and the specimens were identified by specialists at the Jardín Botánico of Quito, Ecuador, using the reference vouchers for specimens found at the center’s herbarium. The edible parts of the samples were chopped and freeze-dried, ground to a fine powder and stored at −20 °C until analysis.

### 2.2. Determination of Total Phenolic, Total Flavonoid, and Total Anthocyanin Contents

The samples were lyophilized and subjected to hydroalcoholic extraction [14]. The total phenolic content (TPC) was determined using the Folin-Ciocalteu method [15] and the results expressed as milligrams of gallic acid equivalents (GAE) per 100 gram of fresh weight (FW) of fruit (mg GAE/100 g FW). The total flavonoid content (TFC) was determined spectrophotometrically [16] and the results expressed as milligrams of catechin equivalents (Cateq) per gram of FW of fruit (mg Cateq/g FW), and total anthocyanin (ACY) content was determined using a modified pH differential method [17], with minor modifications, and the results expressed as milligrams of pelargonidin 3-glucoside equivalents (PgEq) per gram of FW of fruits (mg PgEq/g FW).

### 2.3. Equipment and Conditions for the HPLC-DAD Analysis

The HPLC system (Agilent Technologies Series 1260, Santa Clara, CA, USA), equipped with a quaternary pump (Agilent Technologies 1260 Infinity G1312B, Santa Clara, CA, USA) and a diode array detector (Agilent Technologies 1260 Infinity G1315C DAD, Santa Clara, CA, USA), was used and adjusted according to the specific conditions of each method, as follows.

#### 2.3.1. Vitamin C Content Analysis

Vitamin C was extracted following the procedure previously reported by Alvarez-Suarez et al. [7] and analyzed at 245 nm using the above-described HPLC system. An Eclipse Plus C18 column (5 µm, 4.6 × 150 mm) was used as the stationary phase and KH_2_PO_4_ (50 mM, pH 2.5) was used as a solvent for elution in an isocratic gradient at a flow rate of 1 mL/min for 20 min. Ascorbic acid standard (5 mg/L, y = 235.54x + 53.13, coefficient of determination (*R*^2^) = 0.9975, limit of quantification (LOQ): 0.598 mg/L and limit of detection (LOD): 0.179 mg/L) was used for the calibration curve and total vitamin C content is expressed as mg of ascorbic acid per 100 g of FW of fruits (mg Vit C/100 g FW).

#### 2.3.2. Carotenoid Content Analysis

The carotenoids were extracted and saponified [7] and analyzed at 450 nm using the above-described HPLC system. An Eclipse Plus C18 column (5 µm, 4.6 × 250 mm) was used as the stationary phase and methanol–isopropanol (35:65, *v/v*) was used in an isocratic gradient at a flow rate of 1 mL/min for 15 min for elution. *β*-carotene, lutein, and lycopene contents were quantified using a calibration curve of the corresponding external standard and the results are expressed as mg per 100 g of FW of fruit. 

#### 2.3.3. Organic Acids Content Analysis

Organic acids were extracted according to the method previously reported [18]. A Zorbax SB-Aq column (3.5 µm, 4.6 × 50 mm) was used as the stationary phase and elution was performed with 20 mM of NaH_2_PO_4_ (pH 2.6)/acetonitrile (99.5:0.5, *v/v*) in an isocratic gradient at a flow rate of 0.8 mL/min for 35 min. The detector was set to 210 nm. Lactic, acetic, citric, and oxalic acids were used as standards and the results are expressed as mg or g/100 g of fresh weight (FW) of fruit. 

### 2.4. HPLC-Refractive Index Detector Analysis of Glucose, Fructose, and Sucrose Contents

Glucose and fructose contents were determined according to a previously reported method [19]. The ultra-high performance liquid chromatography (UHPLC) system (Dionex Ultimate 3000, Germering, Germany, Software Chromeleon) coupled with the refractive index (RI) detector was equipped with a quaternary pump (Dionex Ultimate 3000 UHPLC Focussed), degasser, and ERC RI 520 refractive index detector (RefractoMax, 521, Tokyo, Japan), and the chromatographic data were acquired using Chromeleon software. The stationary phase consisted of a Zorbax NH2 (250 × 4.6 mm, 5 µm) and elution was performed with 0.01 N H_2_SO_4_ in an isocratic gradient at a flow rate of 1.0 mL/min for 55 min. External standards of glucose, fructose, and sucrose were used for identification and quantification using the respective calibration curves and the results are expressed as g/100g FW.

### 2.5. Determination of Total Antioxidant Capacity (TAC)

The hydroalcoholic extracts were used for TAC analyses using the ferric reducing antioxidant power (FRAP) assay [20] and the 2,2-diphenyl-1-picrylhydrazyl (DPPH) free radical method [21]. For the FRAP assay, 100 µL of sample or Trolox standard solution was added to 900 µL FRAP reagent (10:1:1 of sodium acetate (300 mM, pH 3.6), TPTZ (10 mM in HCl 40 mM), and ferric chloride (20 mM)). The reaction mixture was vortexed and allowed to stand for 5 min at room temperature in the dark and then the absorbance was read at 593 nm against a blank. 

For the DPPH assay, 100 µL of sample or Trolox standard solution was vigorously mixed with 400 μL of DPPH solution in methanol (0.2 mM). The mixtures were incubated for 15 min at room temperature and the absorbance of the solution (Ab_sol_) was measured at 517 nm. The sample blank (B1) consisted of 600 μL of 70% methanol and 400 μL of DPPH, whereas the fruits blank (B2) contained 100 μL of sample, 500 μLl of 70% methanol, and 400 μL of methanol. The DPPH scavenging activity was calculated using the following equation:

DPPH scavenging activity (%) = [1 − {(Ab_sol_ − B2)/B1}] × 100 (1)

The results are expressed as μmol of Trolox equivalents (TEq) per gram of fresh weight of fruit FW (μmol TEq per g FW) for both assays.

### 2.6. Statistical Analysis

Statistical analyses were performed using IBM SPSS Statistics for Windows version 20.0. A multivariate analysis (MANOVA) was applied to determine the chemical composition and TAC with regards to the fruit species. Variables describing chemical composition and antioxidant properties were treated as dependent variables in the MANOVA and some were previously log transformed for homogeneity fulfillment. The MANOVA analysis was essentially used to test whether or not the fruit type simultaneously explained a statistically significant amount of variance in the chemical composition and antioxidant properties. The Bonferroni correction was used for post-hoc analysis, accounting for comparison between multiples groups. *p* < 0.05 was considered statistically significant. The samples were analyzed in triplicate, and results are reported as mean ± standard deviation (SD).

## 3. Results and Discussion

### 3.1. Total Phenolic, Flavonoid, Anthocyanin, and Vitamin C Contents

The analyzed samples showed significant differences (*p* < 0.05) in the contents of the different bioactive compounds (Table 2). The total phenolic content (TPC) in the different fruits ranged between 149.89 mg (borojo) and 4436.01 mg (carambola) GAE/100 g FW. TPC was also used to classify the fruits into three groups according to the classification previously proposed by Vasco et al. [10]: low (<100 mg of GAE/100 g FW), medium (100–500 mg of GAE/100 g FW), and high contents (>500 mg of GAE/100 g FW). According to this classification, 63.2% of the fruits were classified as having a high TPC and 36.8% were in the medium content category, which is in agreement with the values previously reported in fruits from tropical regions [22,23,24,25]. The total flavonoid content (TFC) ranged between 1.53 (giant granadilla) and 53.28 mg (carambola) CatEq/g FW, where carambola showed the best results for both TPC and TFC assays. In terms of anthocyanin content (ACY), the lowest values were found in borojo fruit (0.24 mg PgEq/g FW), and the highest in chili pepper (ají cacique) (1.35 mg PgEq/g FW).

The values of vitamin C in the fruits analyzed ranged between 2.08 mg of Vit C/100 g of FW (avocado) and 509.63 mg of Vit C/100 g of FW (guava), in line with the results previously reported in different fruits [23,24,26,27] (Table 2). According to the classification proposed by Ramful et al. [28] for the Vit C content in fruits, 64.7% of the fruits analyzed here were classified as high (>50 mg/100 g of FW), and 35.3% as low (<30 mg/100 g of FW). In vanilla and Ecuadorian ivory palm, vitamin C was not detectable in our determination. Following the directives of the Food and Nutrition Board at the Institute of Medicine of the National Academies [29], the recommended dietary allowances (RDAs) for vitamin C for adults over 19 years old are 90 mg for men and 75 mg for women. Therefore, following these recommendations, 58% of the fruits analyzed here exceeded the recommended limits per 100 g of FW, which demonstrates the high content of this vitamin in the fruits consumed in this region.

### 3.2. Total Carotenoid Content

**Figure 1:**
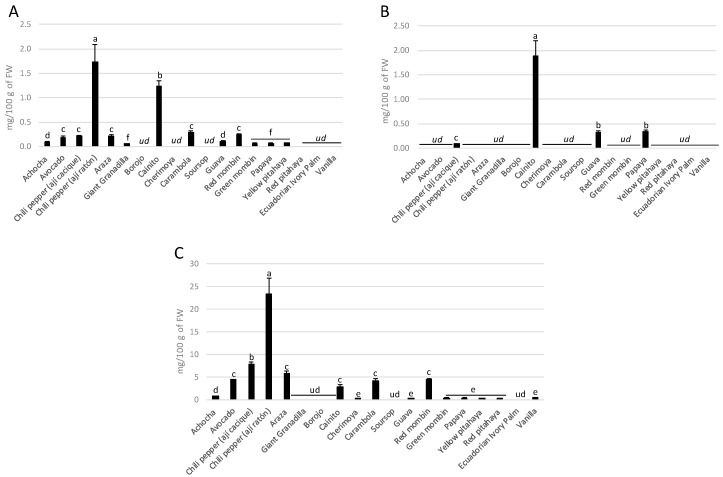
The carotenoid contents in the major fruits consumed in the western coastal region of Ecuador: (**A**) β-carotene, (**B**) lycopene, and (**C**) lutein contents. Values are expressed as means ± SD. Bars with different letters are significantly different per Bonferroni post-hoc analysis (*p* < 0.05). Samples were analyzed in triplicate. *^ud^*—undetectable.

Total carotenoid contents in the fruits analyzed here are shown in Figure 1. The highest concentrations of β-carotene (1.88 mg/100 g of FW) and lutein (22.44 mg/100 g of FW) were found in chili pepper (aji ratón), and cainito fruits were the main source of lycopene (1.87 mg/100 g of FW). Previously, our group reported the carotenoid contents of wild Andean blackberry (*Rubus glaucus* Benth) [13], Andean blueberry (*Vaccinium floribundum* Kunth) [13], and capuli cherry (*Prunus serotina* Ehr. subsp. capuli (Cav.) McVaugh) [7] from the highlands of Ecuador; however, beyond these results, no data were found in the literature regarding the carotenoid content of other fruits from Ecuador. Therefore, we assume this is the first report regarding the carotenoid composition of these fruits. Most of the results reported here are within the range of those values previously reported in fruits from other geographical regions [30,31,32,33,34]. However, in some cases, such as in chili pepper and caimito, the values are higher than those usually reported in other fruits but in line with those previously reported in fruits from the Northern Argentinean Andean region [35]. According to Khayyat et al. [36], the exposure of plants to differences in altitude and the incidence of ultraviolet light, as well as their exposure to low temperatures, has an important effect on their chemical composition. Ecuador is located on the equator, where the sun’s rays strike the earth’s surface more directly (nearly perpendicular or close to a 90° angle) most hours of the day. Therefore, the conditions to which the plants of this region are exposed could justify the high concentrations of carotenoids and other phytocompounds in comparison with those of other regions. Following the intake recommendations for carotenoids [29], all the fruits analyzed here meet the recommendations for daily consumption for this type of compound. 

### 3.3. Organic Acids Contents

Organic acids play an important role in fruit in terms of their texture and flavor, which is fundamental to consumer acceptance [37]. In this context, the total contents of lactic, acetic, citric, and oxalic acids were determined in the fruits (Table 3). The highest lactic acid value was found in soursop fruits (13.56 g/100 g of FW); Ecuadorian ivory palm (8.01 g/100 g of FW) and guava fruits (7.39 g/100 g of FW) showed the highest acetic acid content. 

Guava also had the highest content of citric acid (2.12 g/100 g of FW), whereas the highest concentration of oxalic acid was found in carambola (2.15 g/100 g of FW). We must emphasize that it was difficult to find reports on the content of organic acids in the fruits produced both in this region and in Ecuador; therefore, in our opinion, the results reported here constitute the first study regarding the profile of organic acids in fruits from Ecuador. We compared our results with those previously reported in fruits from other geographical areas and it was found that the values reported here are within the ranges previously described [18,37]. Although the values reported here could be compared with those from previous reports, it should be taken into account that the organic acid content depends on the fruit’s stage of development and on different environmental and storage factors [36,38], allowing one to justify the differences between our results and those previously reported in fruits from other geographical regions.

### 3.4. Sugar Content

Fruits and vegetables naturally contain sucrose, glucose, and fructose, among other carbohydrates in varying amounts, and their contents directly affect the quality and flavor of fruits [19]. Sugars found in fruits and vegetables are considered healthier than added sugars, and therein lies the importance of knowing the main natural sources for each, to incorporate them into one’s diet. The glucose, fructose, and sucrose contents in the fruits analyzed are presented in Table 4. Giant granadilla showed the highest values of glucose (5.28 g/100 g of FW); red mombin was the principal source of fructose (7.22 g/100 g of FW), and guava of sucrose (7.40 g/100 g of FW). However, considering the sum of these three sugars, red mombin showed the highest values, followed by guava, whereas the lowest values were found in the fruits of green mombin, carambola, and Ecuadorian ivory palm (Table 4). These results are within the range of those previously reported in fruits from other geographical regions; however, these values could also be considered low in comparison with those previously reported [27], which could be considered positive if considering the negative effect of excess sugar consumption on health. The RDA [39] established for carbohydrate is 130 g per day for adults and children. Following these recommendations, all the fruits analyzed could be considered low in carbohydrate contributions for 100 g of FW. In this sense, 100 g of the fruits of red mombin, which were those that showed the highest total sugar content (11.04 g/100 of FW) (glucose + fructose + sucrose), as the main sugars contributed by fruits, would provide approximately only 8.5% of the total recommended carbohydrates for a day (130 g of total carbohydrates).

### 3.5. Total Antioxidant Activity

**Figure 2:**
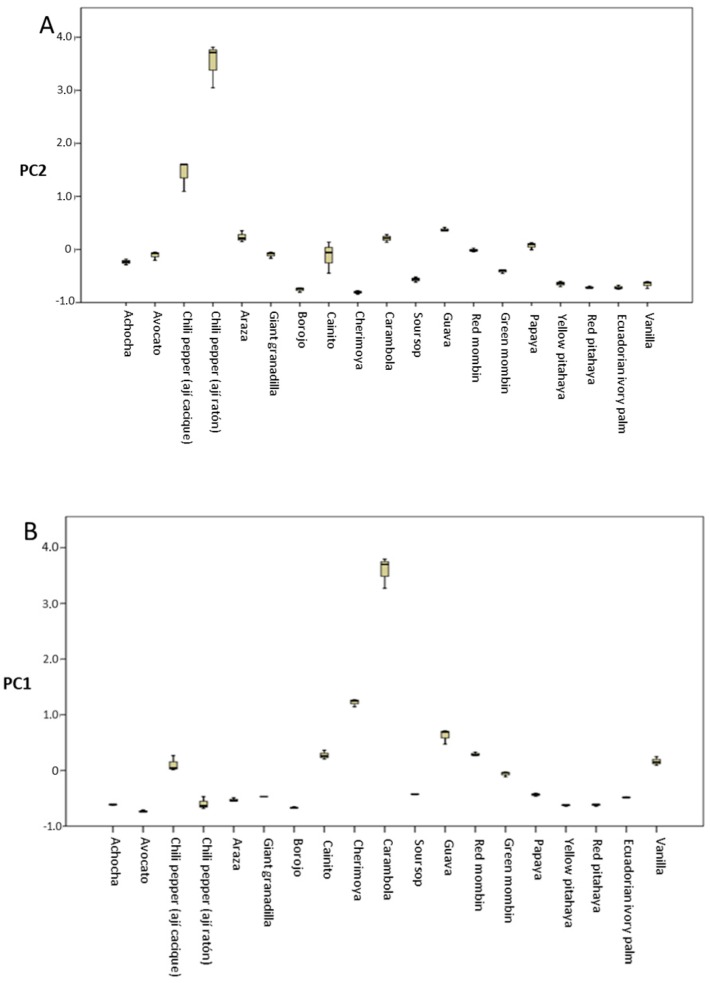
Factorial analysis and cluster using varimax rotation with the TFC, TPC, FRAP, DPPH, Lut, β-carot Lut, ACY, and Vit C values. The distributions of (**A**) PC1 and (**B**) PC2. FRAP—ferric reducing antioxidant power; DPPH—2,2-diphenyl-1-picrylhydrazyl free radical method; TPC—total phenolic content; TFC—total flavonoid content; Vit C—vitamin C content; ACY—total anthocyanin content; *β*-Carot—total β-carotene content; Licp—total lycopene content; Lut—total lutein content.

In relation to the total antioxidant capacity (TAC), the highest values for both methods (FRAP, DPPH) were found in carambola (Table 2). In addition, given that the fruits’ health benefits are influenced by the interaction of their different bioactive compounds, a factorial analysis was conducted with the principal variables (TFC, TPC, FRAP, DPPH, ACY, *β*-carotene, lutein, and Vit C) related to the TAC, using the varimax rotation to determine which fruit was the main contributor for of each of them. Three factors were extracted (principal component (PC) PC1, PC2, and PC3), which explained 77.46% of the variance. The rotated matrix clearly described the main contributions of each component. The first two components explained 64.78% of the total variance and encompassed the majority of the variables (Table 5), while Figure 2 shows the distribution of the PC1 and PC2 variables. Carambola was found to be the main contributor in terms of TPC and TFC, and TAC (Figure 2A), whereas chili pepper (ají ratón) proved to be the main source of lutein, β-carotene, as well as ACY, and Vit C (Figure 2B). The reason that carambola had the highest results for TAC could be explained by its TPC and TFC, which seem to be the principal contributors to the TAC in the fruits analyzed here (Table 2). Indeed, the correlation analysis clearly shows that FRAP and DPPH are highly correlated with TPC and TFC. Moreover, FRAP but not DPPH is related to vitamin C, ACY, and other compounds with antioxidant effects, which may in turn be related to the antioxidant mechanisms of the compounds present in these fruits, which may have more reducing activity (FRAP) than radical scavenging (DPPH) activity (Table 6). Therefore, although Vit C and carotenoids have been associated with important antioxidant activity in fruits [40], according to the high correlation found here between TFC, TPC, FRAP, and DPPH, it could be hypothesized that polyphenols could be mainly responsible for the TAC in the fruits studied here, in accordance with previous reports [22,34].

## 4. Conclusions

In this study, selected bioactive compounds and antioxidant capacity were determined for the main fruits consumed in Ecuador’s western coastal region, with the aim of elucidating their potential as dietary sources of bioactive compounds with beneficial effects on human health. Carambola is the main contributor in terms of the total phenolic and flavonoid contents and total antioxidant capacity, whereas chili pepper (ají ratón) is the main contributor of lutein, β-carotene, vitamin C, and anthocyanins. The correlation analysis showed that the total phenolic and flavonoid contents are the main contributors to the total antioxidant capacity of the fruits. In general, the results presented here, to the best of our knowledge, constitute the first report of the contents of important bioactive compounds with relevant effects on human health, as well as other compounds that are related to the quality of these exotic fruits from Ecuador. The results presented here contribute to determining the potential of these tropical fruits as sources of biological compounds beneficial to human health, potentially justifying their consumption as new sources of these types of compounds for human nutrition. 

## Figures and Tables

**Table 1:** Major fruits consumed in the western coastal region of Ecuador.

Local Name	Common Name	Scientific Name	Type	Status
Achogchas	Achocha/caihua	*Cyclanthera pedata* (L.) Schrad.	Vine	Native and cultivated
Aguacate	Avocado	*Persea americana* Mill.	Tree	Cultivated
Ají cacique	Chili pepper	*Capsicum annuum L.*	Herb	Cultivated
Ají ratón	Chili pepper	*Capsicum chinense* Jacq	Herb	Cultivated
Arazá	Araza/araca boi	*Eugenia stipitata* McVaugh	Shrub	Native and cultivated
Badea	Giant granadilla, badea	*Passiflora quadrangularis* L.	Vine or liana	Cultivated
Borojó	Borojo	*Borojoa patinoi* Cuatrec.	Tree	Introduced
Caimito	Cainito/tar apple/star apple	*Chrysophyllum cainito* L.	Tree	Introduced
Chirimoya	Cherimoya/custard apple	*Annona cherimola* Mill	Shrub	Native and cultivated
Fruta china	Carambola/star fruit	*Averrhoa carambola* L.	Tree	Introduced
Guanábana	Soursop	*Annona muricata* L.	Tree	Native and cultivated
Guayaba	Guava	*Psidium guajava* L.	Shrub	Native and cultivated
Hobo rojo	Red mombin	*Spondias mombin* L.	Tree	Native
Hobo verde	Green mombin	*Spondias mombin* L.	Tree	Native
Papaya	Papaya	*Carica papaya* L.	Tree	Native and cultivated
Pitahaya amarilla	Yellow pitahaya/yellow dragon fruit	*Cereus megalanthus* K. Schum. ex Vaupel	Epiphyte	Native
Pitahaya roja	Red pitahaya/strawberry pear	*Cereus ocamponis* Salm-Dyck	Epiphyte	Introduced
Tagua	Ecuadorian ivory palm	*Phytelephas aequatorialis* Spruce	Tree	Endemic
Vainilla	Vanilla	*Vanilla planifolia* Andrews	Epiphyte	Native and cultivated

**Table 2:** Chemical composition and antioxidant capacity of the major fruits consumed in the western coastal region of Ecuador.

Common Name	TAC (µmol TEq/g FW)		Bioactive Compounds			
	FRAP	DPPH	TPC (mg GAE/100 g FW)	TFC (mg CatEq/g FW)	ACY (mg PgEq/g FW)	Vit C (mg Vit C/100 g FW)
Achocha	264.78 ± 21.14 ^e^	0.24 ± 0.05 ^i^	482.07 ± 48.99 ^d^	2.88 ± 0.23 ^e^	0.68 ± 0.07 ^b^	18.03 ± 4.07 ^f^
Avocado	189.09 ± 37.31 ^e^	*^ud^*	317.02 ± 10.42 ^d^	2.50 ± 0.63 ^e^	0.42 ± 0.09 ^c^	1.63 ± 0.43 ^g^
Chili pepper	1666.66 ± 139.00 ^b^	91.47 ± 5.56 ^e^	1282.15 ± 352.90 ^b^	7.40 ± 1.17 ^c^	1.33 ± 0.26 ^a^	248.13 ± 56.53 ^c^
Chili pepper	923.05 ± 53.85 ^c^	85.49 ± 5.97 ^e^	1184.67 ± 199.85 ^b^	3.61 ± 0.38 ^d^	0.76 ± 0.07 ^b^	294.00 ± 27.36 ^c^
Araza	24.56 ± 5.35 ^h^	210.78 ± 16.50 ^d^	543.32 ± 21.94 ^d^	1.84 ± 0.46 ^e^	0.32 ± 0.04 ^c^	181.13 ± 13.01 ^d^
Giant Granadilla	387.89 ± 33.11 ^d^	90.68 ± 8.05 ^e^	324.18 ± 36.35 ^d^	1.79 ± 0.24 ^e^	0.33 ± 0.02 ^c^	277.72 ± 22.24 ^c^
Borojo	3.66 ± 0.92 ^j^	50.07 ± 8.18 ^g^	142.53 ± 16.20 ^e^	3.88 ± 0.29 ^d^	0.30 ± 0.05 ^c^	*^ud^*
Cainito	1591.93 ± 398.06 ^b^	537.61 ± 43.81 ^c^	1181.44 ± 172.13 ^b^	8.25 ± 0.37 ^c^	0.83 ± 0.10 ^b^	12.23 ± 1.61 ^f^
Cherimoya	1167.18 ± 183.84 ^c^	843.80 ± 19.28 ^b^	1472.18 ± 332.49 ^b^	23.43 ± 5.00 ^b^	0.27 ± 0.05 ^c^	78.96 ± 6.13 ^e^
Carambola	3370.94 ± 308.02 ^a^	1215.34 ± 101.98 ^a^	4280.83 ± 673.83 ^a^	48.52 ± 5.40 ^a^	0.51 ± 0.07 ^b^	199.44 ± 11.28 ^d^
Soursop	422.11 ± 57.72 ^d^	92.96 ± 9.46 ^e^	485.85 ± 36.38 ^d^	2.15 ± 0.18 ^e^	0.21 ± 0.06 ^c^	106.00 ± 8.50 ^d^
Guava	1568.48 ± 273.20 ^b^	607.48 ± 18.96 ^c^	1163.94 ± 159.50 ^b^	5.80 ± 0.53 ^c^	0.57 ± 0.05 ^b^	496.73 ± 14.32 ^a^
Red mombin	45.31 ± 6.85 ^g^	1081.79 ± 50.18 ^a^	827.51 ± 14.58 ^c^	4.39 ± 0.34 ^d^	0.35 ± 0.03 ^c^	201.02 ± 17.93 ^d^
Green mombin	46.63 ± 8.39 ^g^	519.18 ± 30.99 ^c^	787.87 ± 38.87 ^c^	4.40 ± 0.49 ^d^	0.33 ± 0.03 ^c^	176.25 ± 14.03 ^d^
Papaya	571.26 ± 59.62 ^d^	77.77 ± 8.35 ^f^	341.70 ± 42.97 ^d^	1.99 ± 0.48 ^e^	0.54 ± 0.04 ^b^	341.98 ± 20.22 ^b^
Yellow pitahaya	9.04 ± 1.30 ^i^	31.91 ± 4.40 ^h^	389.74 ± 39.49 ^d^	3.57 ± 0.3 ^d^	0.33 ± 0.07 ^c^	4.80 ± 0.58 ^g^
Red pitahaya	10.53 ± 2.30 ^i^	84.61 ± 3.89 ^e^	251.22 ± 39.04 ^e^	3.41 ± 0.34 ^d^	0.32 ± 0.02 ^c^	12.93 ± 2.52 ^f^
Ecuadorian ivory palm	381.85 ± 46.36 ^d^	116.06 ± 7.55 ^d^	203.54 ± 20.008 ^e^	4.50 ± 0.34 ^d^	0.34 ± 0.06 ^c^	*^ud^*
Vanilla	78.86 ± 9.64 ^f^	530.32 ± 22.88 ^c^	1447.25 ± 171.05 ^b^	6.71 ± 0.66 ^c^	0.52 ± 0.09 ^b^	*^ud^*

Note: Values are expressed as means ± SD. Mean values within a column sharing the same letter are not significantly different after Bonferroni post-hoc analysis (*p* < 0.05). Each sample was analyzed in triplicate. *^ud^*—undetectable; FRAP—ferric reducing antioxidant power; DPPH—2,2-diphenyl-1-picrylhydrazyl free radical method; TPC—total phenolic content; TFC—total flavonoid content; ACY—total anthocyanin content; Vit C—vitamin C content.

**Table 3:** Contents of organic acids in the principal fruits consumed in the western coastal region of Ecuador.

Common Name	Organic Acids (mg/100 g of FW)			
	Lactic Acid	Acetic Acid	Citric Acid	Oxalic Acid
Achocha	1.85 ± 0.01 ^c^	1.66 ± 0.02 ^b^	0.47 ± 0.05 ^c^	0.04 ± 0.00 ^e^
Avocado	2.01 ± 0.30 ^c^	2.83 ± 0.16 ^b^	0.03 ± 0.00 ^e^	0.05 ± 0.00 ^e^
Chili pepper	1.65 ± 0.13 ^c^	0.76 ± 0.02 ^c^	1.88 ± 0.04 ^a^	0.05 ± 0.00 ^e^
Chili pepper	4.44 ± 0.71 ^b^	0.59 ± 0.03 ^c^	0.42 ± 0.05 ^c^	0.02 ± 0.00 ^e^
Araza	5.18 ± 0.07 ^b^	0.53 ± 0.01 ^c^	0.11 ± 0.00 ^d^	*^ud^*
Giant Granadilla	0.56 ± 0.03 ^e^	1.29 ± 0.09 ^b^	0.90 ± 0.01 ^b^	0.13 ± 0.00 ^d^
Borojo	1.48 ± 0.17 ^c^	0.12 ± 0.01 ^d^	0.02 ± 0.00 ^e^	0.58 ± 0.05 ^b^
Cainito	1.32 ± 0.01 ^c^	1.65 ± 0.07 ^b^	0.04 ± 0.00 ^e^	0.21 ± 0.01 ^c^
Cherimoya	1.12 ± 0.03 ^d^	1.57 ± 0.01 ^b^	0.53 ± 0.00 ^c^	0.01 ± 0.00 ^e^
Carambola	0.93 ± 0.04 ^d^	0.67 ± 0.06 ^c^	0.02 ± 0.00 ^e^	2.15 ± 0.11 ^a^
Soursop	13.56 ± 2.00 ^a^	1.42 ± 0.24 ^b^	0.41 ± 0.02 ^c^	0.02 ± 0.00 ^e^
Guava	0.92 ± 0.06 ^d^	7.39 ± 0.64 ^a^	2.12 ± 0.26 ^a^	0.06 ± 0.00 ^e^
Red mombin	0.64 ± 0.03 ^e^	1.54 ± 0.04 ^b^	0.12 ± 0.01 ^d^	0.02 ± 0.00 ^e^
Green mombin	0.41 ± 0.05 ^e^	1.39 ± 0.03 ^b^	0.09 ± 0.01 ^d^	*^ud^*
Papaya	1.82 ± 0.36 ^c^	0.08 ± 0.00 ^d^	1.10 ± 0.02 ^b^	0.23 ± 0.01 ^c^
Yellow pitahaya	0.58 ± 0.04 ^e^	0.13 ± 0.03 ^d^	0.88 ± 0.01 ^b^	0.07 ± 0.00 ^e^
Red pitahaya	2.12 ± 0.02 ^c^	*^ud^*	0.08 ± 0.00 ^d^	0.14 ± 0.01 ^d^
Ecuadorian ivory palm	4.66 ± 0.18 ^b^	8.01 ± 0.38 ^a^	0.11 ± 0.01 ^d^	0.02 ± 0.00 ^e^
Vanilla	0.07 ± 0.01 ^f^	0.02 ± 0.00 ^e^	1.98 ± 0.73 ^a^	0.02 ± 0.00 ^e^

Values are expressed as means ± SD. Mean values within a column with a different letter are significantly different after Bonferroni post-hoc analysis (*p* < 0.05). Samples were analyzed in triplicate. *^ud^*—undetectable.

**Table 4:** Sugar contents in the principal fruits consumed in the western coastal region of Ecuador.

Common Name	Sugar Content (g/100 g of FW)			
	Glucose	Fructose	Sucrose	Total ∑ _Gluc+Fruct+Sucr_
Achocha	2.04 ± 0.32 ^c^	2.12 ± 0.20 ^c^	1.80 ± 0.62 ^c^	5.97 ± 0.26 ^c^
Avocado	0.56 ± 0.04 ^d^	4.86 ± 0.23 ^b^	0.28 ± 0.09 ^e^	5.70 ± 1.56 ^c^
Chili pepper	*^ud^*	0.06 ± 0.01 ^g^	0.41 ± 0.02 ^e^	0.47 ± 0.12 ^f^
Chili pepper	0.02 ± 0.00 ^f^	4.47 ± 0.71 ^b^	0.60 ± 0.01 ^d^	5.10 ± 1.42 ^c^
Araza	0.54 ± 0.02 ^d^	0.18 ± 0.04 ^f^	0.07 ± 0.00 ^f^	0.80 ± 0.14 ^f^
Giant Granadilla	5.88 ± 0.67 ^a^	0.26 ± 0.03 ^f^	0.52 ± 0.08 ^d^	6.66 ± 1.79 ^c^
Borojo	2.82 ± 0.10 ^b^	1.84 ± 0.16 ^c^	0.27 ± 0.03 ^e^	4.93 ± 1.27 ^c^
Cainito	0.04 ± 0.00 ^f^	1.60 ± 0.06 ^c^	1.63 ± 0.07 ^c^	3.27 ± 0.84 ^d^
Cherimoya	3.40 ± 0.38 ^b^	0.51 ± 0.11 ^e^	0.05 ± 0.00 ^f^	3.96 ± 1.27 ^d^
Carambola	0.65 ± 0.13 ^d^	0.64 ± 0.11 ^e^	0.04 ± 0.00 ^f^	1.33 ± 0.35 ^e^
Soursop	0.42 ± 0.01 ^d^	0.67 ± 0.05 ^e^	1.92 ± 0.06 ^c^	3.01 ± 0.80 ^d^
Guava	0.15 ± 0.02 ^e^	0.92 ± 0.01 ^d^	7.40 ± 0.65 ^a^	8.47 ± 2.18 ^b^
Red mombin	1.65 ± 0.17 ^c^	7.22 ± 0.08 ^a^	2.17 ± 0.22 ^c^	11.04 ± 2.94 ^a^
Green mombin	1.51 ± 0.27 ^c^	0.17 ± 0.02 ^f^	0.08 ± 0.00 ^f^	1.76 ± 0.80 ^e^
Papaya	1.48 ± 0.34 ^c^	0.71 ± 0.04 ^d^	3.45 ± 0.29 ^b^	5.64 ± 1.40 ^c^
Yellow pitahaya	2.81 ± 0.13 ^b^	0.82 ± 0.10 ^d^	0.75 ± 0.04 ^d^	4.38 ± 1.17 ^c^
Red pitahaya	*^ud^*	0.21 ± 0.01 ^f^	2.21 ± 0.18 ^c^	2.42 ± 0.82 ^d^
Ecuadorian ivory palm	0.46 ± 0.06 ^d^	0.86 ± 0.03 ^d^	0.02 ± 0.00 ^f^	1.34 ± 0.42 ^e^
Vanilla	0.17 ± 0.02 ^e^	0.51 ± 0.06 ^e^	1.48 ± 0.68 ^c^	2.16 ± 0.67 ^d^

Note: Values are expressed as means ± standard deviation (SD). Mean values within a column with different letters are significantly different after Bonferroni post-hoc analysis (*p* < 0.05). Samples were analyzed in triplicate. *^ud^*—undetectable.

**Table 5:** Rotated coupon in factorial analysis.

Variable	PC1	PC2	PC3
TFC	0.951	−0.014	−0.037
TPC	0.939	0.218	0.025
FRAP	0.836	0.322	0.262
DPPH	0.835	−0.090	−0.002
Lutein	−0.015	0.935	−0.029
B-Carotene	0.003	0.774	0.441
ACY	0.091	0.604	0.469
Vit C	0.218	0.546	−0.203
Licp	0.072	0.002	0.952

Note: FRAP—ferric reducing antioxidant power; DPPH—2,2-diphenyl-1-picrylhydrazyl free radical method; TPC—total phenolic content; TFC—total flavonoid content; Vit C—vitamin C content; ACY—total anthocyanin content; *β*-Carot—total *β*-carotene content; Licp—total lycopene content; Lut—total lutein content.

**Table 6:** Correlation matrix for chemical composition and total antioxidant capacity (TAC) in the fruits analyzed.

Variables	FRAP	DPPH	TPC	TFC	Vit C	ACY	β-Carot	Licp	Lut
FRAP	1	0.526 **	0.838 **	0.797 **	0.366 **	0.449 **	0.293 *	0.294 *	0.217
DPPH		1	0.719 **	0.699 **	0.201	−0.084	0.012	0.126	−0.047
TPC			1	0.908 **	0.210	0.250	0.197	0.058	0.214
TFC				1	0.045	0.049	0.016	−0.015	0.025
Vit C					1	0.261 *	0.168	0.001	0.295 *
ACY						1	0.437 **	0.345 **	0.445 **
*β*-Carot							1	0.433 **	0.821 **
Licp								1	−0.050
Lut									1

** Correlation significant at the 0.01 level; * Correlation significant at the 0.05 level. FRAP—ferric reducing antioxidant power; DPPH—2,2-diphenyl-1-picrylhydrazyl free radical method; TPC—total phenolic content; TFC—total flavonoid content; Vit C—vitamin C content; ACY—total anthocyanin content; *β*-Carot—total *β*-carotene content; Licp—total lycopene content; Lut—total lutein content.
